# CineECG detects abnormal electrical activity in the 12-lead ECG of preclinical plakophilin-2 variant carriers

**DOI:** 10.1016/j.hroo.2025.07.105

**Published:** 2025-08-08

**Authors:** Iris van der Schaaf, Manon Kloosterman, Anton P.M. Gorgels, Anneline S.J.M. te Riele, Peter M. van Dam, Peter Loh

**Affiliations:** 1 Department of Cardiology, University Medical Center Utrecht, Utrecht, The Netherlands; 2 European Reference Network for Rare and Low Prevalence Complex Diseases of the Heart (ERN GUARD-Heart), The Netherlands; 3 Department of Cardiology, Maastricht University Medical Center, Maastricht, The Netherlands; 4 Center for Digital Medicine and Robotics, Jagiellonian University Medical College, Kopernika, Kraków, Poland; 5 3D Functional and Virtual Medical Imaging Laboratory, Department of Diagnostic Imaging, University Hospital in Krakow, Krakow, Poland

**Keywords:** Arrhythmogenic cardiomyopathy, CineECG, Electrocardiogram, Genetics, Electrophysiology

## Abstract

**Background:**

Carriers of (likely) Plakophilin-2 pathogenic variants (*PKP2*-(L)PV) are at risk of developing arrhythmogenic cardiomyopathy. Early disease detection is crucial because life-threatening arrhythmias may occur early. CineECG is a novel electrocardiogram (ECG) analysis tool that reconstructs the average trajectory of ventricular electrical activity.

**Objective:**

The study aimed to describe the electrical depolarization and repolarization CineECG trajectories in *PKP2*-(L)PV carriers with a normal ECG as per evaluation of 2 cardiologists, who meet no Task Force Criteria other than their PV.

**Methods:**

*PKP2*-(L)PV carriers were 2:1-matched to control subjects, who had atrioventricular nodal reentry tachycardia but no other cardiac abnormalities. Sinus rhythm ECGs of controls were used to create a normal distribution of trajectories. *PKP2*-(L)PV carriers’ trajectories were compared with the normal distribution. A trajectory was considered abnormal if it fell less than 95% within the normal distribution.

**Results:**

Overall, 104 subjects were included (age 24 years [19–36], 43% men): 37 *PKP2*-(L)PV carriers and 67 controls. Depolarization and repolarization trajectories were abnormal in 51% and 24% of carriers, respectively. In carriers with abnormal depolarization trajectories, significant differences were observed in the direction of the initial depolarization trajectory when compared with controls in the inferior-superior axis (*P* = .005) and posterior-anterior axis (*P* = .020). In the left-right axis, the direction significantly differed from carriers with a normal trajectory (*P* = .020).

**Conclusion:**

Abnormal electrical activity was identified in over half of preclinical *PKP2*-(L)PV carriers with a normal ECG. CineECG could be a sensitive tool to unveil early, subtle abnormalities in ventricular electrical activity that would otherwise not be detected.

**Key Findings**

▪ The average depolarization and repolarization pathways throughout the heart were identified in preclinical plakophilin-2 carriers using only the 12-lead electrocardiogram, which revealed abnormal depolarization in 51% of the carriers and abnormal repolarization in a 24% of the carriers.
▪ Carriers with an abnormal depolarization trajectory showed greatest differences in the direction of the initial part of the depolarization compared to controls.
▪ No distinct differences in the direction of the repolarization trajectory were observed between carriers and controls.

## Introduction

CineECG is a newly developed diagnostic technique through which cardiac depolarization and repolarization patterns can be obtained in a non-invasive manner. Based on the 12-lead electrocardiogram (ECG), CineECG estimates the average location of the depolarization and repolarization throughout the cardiac cycle, resulting in a depolarization and repolarization trajectory which is projected on a 3D-heart model. The methodology of the CineECG trajectory is based on the vectorcardiogram, although main differences are that CineECG allows for electro-anatomical correlation and that the amplitude of the ECG-signal is not part of the CineECG calculation.^1^ This is hypothesized to make the CineECG more sensitive to abnormalities in ventricular de- and repolarization, as minor changes in amplitude are more easily visualized.

Other diagnostic tools have been described to non-invasively estimate the electrical activity of the heart, although they mostly rely on the use of an extensive amount of electrodes and are computationally time-consuming.^2^ CineECG might be an alternative that can be easily implemented in clinical practice, as only a 12-lead ECG is needed which can be analyzed retrospectively. The CineECG trajectory analysis has previously been applied in the identification and classification of conduction disorders, early visualization of ECG changes in myocardial ischemia, and the identification of abnormal electrical activation in Brugada syndrome and familial ST-segment depression syndrome.^3^, ^4^, ^5^, ^6^, ^7^

Although a very promising tool, CineECG has not yet been applied to establish the ventricular electrical activity in arrhythmogenic cardiomyopathy (ACM). This inherited disease is characterized by the occurrence of heart failure and arrhythmias. Diagnosis is determined by the Task Force Criteria (TFC); a combination of diagnostic criteria based on multiple modalities. The presence of genetic variants in genes such as plakophilin-2 (*PKP2*) is an important criterion within the TFC.^8^^,^^9^ Once ACM has been diagnosed and a pathogenic variant is discovered, it is advised that family members are genetically screened to improve detection of disease at an early stage, which has led to an increasing number of subjects who are undergoing cardiological evaluations at a preclinical stage.^10^ As ventricular arrhythmias and even sudden cardiac arrest may be the first signs of disease, it remains of great importance to find early markers of disease expression to aid follow-up and risk stratification.^11^ CineECG might provide additional insight into early electrical abnormalities in this population.

We aim to describe the electrical activity (ie, depolarization and repolarization trajectories) in preclinical carriers of (likely) pathogenic *PKP2*-variants (*PKP2*-(L)PV) using CineECG. We hypothesize that CineECG can detect abnormal depolarization and repolarization in patients with ECGs that are clinically classified as normal.

## Methods

### Study design and population

This was a retrospective cohort study performed at the University Medical Center Utrecht. Study subjects above age 14 were included if genetic testing revealed a *PKP2*-(L)PV as per American College of Medical Genetics criteria.^12^ Otherwise, none of the subjects fulfilled any other TFC for ACM, as determined by clinical evaluation at baseline which at minimum included 12-lead ECG, Holter monitoring, and imaging (echocardiography and/or cardiac magnetic resonance imaging [CMR]), and did not have a history of ventricular tachycardia. All *PKP2*-(L)PV subjects were matched in a 2:1 ratio to an age- and sex-matched control group. Age matching allowed for a difference of up to 5 years in age. Control subjects were subjects who were referred for atrioventricular nodal reentry tachycardia ablation who had an ECG with sinus rhythm available prior to ablation, a structurally normal heart as determined by echocardiography and/or CMR, and no other history of heart disease. Control subjects that used anti-arrhythmic drugs at the time of the ECG were excluded from the control group.

All subjects were part of The Netherlands Arrhythmogenic Cardiomyopathy Registry (www.acmregistry.nl, UCC-UNRAVEL #12-387), or the QRS-VISION/STT-VISION projects (#17-907, #22-796). This study was conducted according to the Declaration of Helsinki and the study protocols were approved by the local institutional ethics review board. Oral and written informed consent was obtained from all the subjects who were a part of the UCC-UNRAVEL/QRS-VISION projects. The STT-VISION project received approval from the institutional ethics review board to waive informed consent in specific cases that involved only retrospective de-identified ECG data.

### Additional evaluation and follow-up

For all *PKP2*-(L)PV subjects, Holter and imaging (echocardiography and/or CMR) performed at baseline were additionally evaluated for the presence of any mild structural and/or electrical abnormalities not fulfilling TFC to assess correlation with abnormally classified CineECG trajectories. A mild abnormality on imaging was defined as: wall motion abnormalities, a mildly reduced left or right ventricular (RV) ejection fraction, and/or unbalanced left ventricular/RV volumes, as described in the report of the treating radiologist/cardiologist. Additionally, CMR specific mild abnormalities included presence of minimal delayed gadolinium enhancement and/or fatty infiltration. For echocardiography, a mild abnormality could also be assumed if an abnormal deformation pattern was found, as per previously published criteria.^13^ Holter data were classified to show a mild abnormality if >100 but <500 premature ventricular contractions were present within 24 hours of Holter monitoring.

Furthermore, follow-up data were collected to determine if abnormal CineECG trajectories corresponded with acquiring new TFC, symptoms of palpitations, syncope, ventricular tachycardia (VT), or implantable cardioverter-defibrillator implantation during follow-up. Follow-up included all clinical data that were available from the patient record, including 12-lead ECGs, Holter monitoring and/or imaging (echocardiography or CMR) that were performed after baseline.

### ECG analysis

The 12-lead ECGs were recorded as part of standard clinical practice. ECGs were evaluated by 2 cardiologists for depolarization and repolarization TFC, and for abnormalities outside of the TFC. All baseline ECGs were transformed to CineECG (version 0.1.0.6882, ECG Excellence BV, The Netherlands). With CineECG, the average location of ventricular electrical activity was estimated throughout the cardiac cycle based on the 12-lead ECG signals, creating a trajectory through the ventricles which was visualized on a 3D-heart model of a healthy 22-year-old subject. An elaborate description on the CineECG trajectory calculation was previously published.^1^ The depolarization (defined as QRS-onset until J-point) and repolarization (J-point until end of T-wave) were separately analyzed, atrial activation was disregarded in this study. The indices for QRS-onset, J-point and T-end, were manually annotated.

### CineECG trajectory analysis

#### Comparison to control subjects

**Figure 1:**
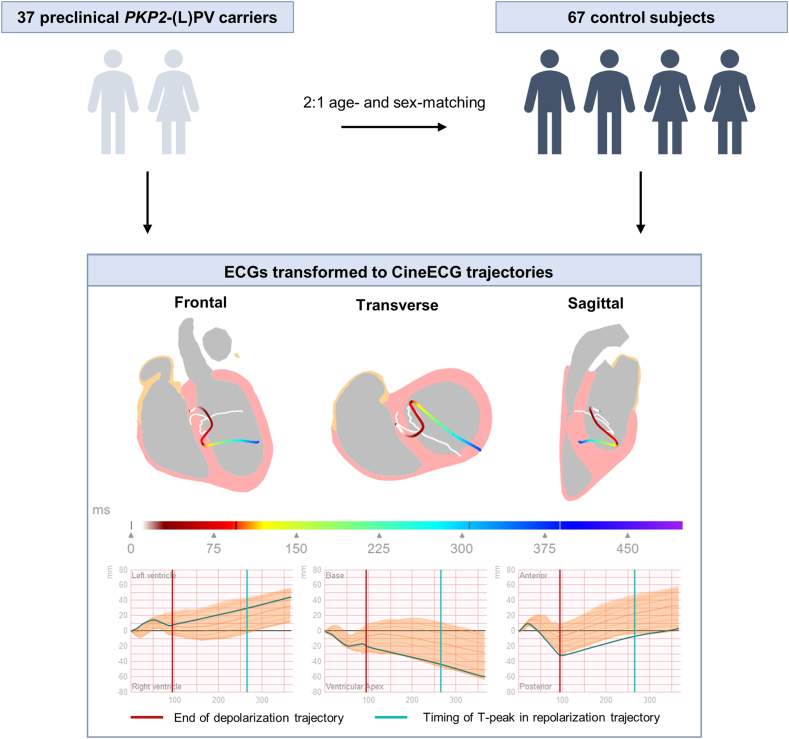
Flowchart. After 2:1 age- and sex-matching the preclinical Plakophilin-2 (likely) pathogenic variant (*PKP2*-(L)PV) carriers to control subjects, the ECGs were transformed to CineECG trajectories. ECGs of control subjects were used to create a normal distribution of trajectories. The median of this normal distribution is shown entirely in *white*. An example of a trajectory of a *PKP2*-(L)PV carrier is shown in color (start depolarization = *white*, end repolarization = *blue*). The lower row demonstrates the direction of the trajectory of the *PKP2*-(L)PV carrier (*green line*) and normal distribution (*orange*) as seen from 3 different axes.ECG = electrocardiogram.

The ECGs of the control subjects were used to compute a normal distribution of CineECG trajectories (Figure 1). For the normal distribution, the starting point of the CineECG trajectories were aligned and the direction of the trajectory was determined along 3 anatomical axes (left-right, inferior-posterior, and posterior-anterior) for each time sample. The upper and lower 1% were removed from the distribution to remove any outliers. Hereafter, the percentage of each trajectory of the *PKP2*-(L)PV carriers that fell within this normal distribution was determined separately for the depolarization and the repolarization trajectory (Figure 1). The ECGs of the *PKP2*-(L)PV subjects were divided into 2 groups based on the amount of the depolarization and repolarization trajectory that fell within the normal distribution. If less than 95% of either trajectory was within the normal distribution, it was considered abnormal. If 95% or more of the trajectory was within the normal distribution, it was considered to be normal. This threshold was selected to allow for minor differences between variant carriers and controls, while still maintaining a relatively strict cut-off value to detect subtle but potentially meaningful differences.

#### Directions

The direction of the trajectory was determined for the initial depolarization trajectory (defined as first 25 ms of the QRS-complex to approach approximately the first third of the normal QRS-complex), terminal depolarization trajectory (defined as 20 ms before the end of the QRS-complex until 30 ms after the end of the QRS-complex, which will include the latest part of depolarization and may also include possible late activation taking place after the J-point index), the part of the repolarization trajectory representing the ST-segment (end of QRS-complex until peak T-wave), and the part of the repolarization trajectory representing the terminal T-wave (peak T-wave until the end of the T-wave).

Directions were assessed along the 3 anatomical axes. For each axis, values were set to range between −1 and 1, where 0 indicates the origin of the directional vector. The directions were then divided into 3 categories based on their position along each axis: values between −0.5 and 0.5 were considered central, values beyond these thresholds were classified as far left or far right, and far inferior/posterior or far posterior/anterior (see Supplemental Figure 1). The directions were compared between the control group and the *PKP2*-(L)PV groups with normal and abnormal depolarization and repolarization trajectories separately.

### Statistical analysis

Analysis was performed in SPSS Statistics (version 29.0.1, IBM Corp, Armonk, New York) and R Statistical Software (version 2022.07.2, R Foundation for Statistical Computing, Vienna, Austria). Categorical data were reported as numbers (percentages). Continuous variables were reported as mean ± standard deviation when normally distributed, and as median with interquartile range (IQR) when non-normally distributed. Data was tested for normality using the Shapiro-Wilk test. Differences between continuous variables were tested for significance using the independent samples t-test for comparing 2 means for normally distributed data, and the Mann-Whitney U test for non-normally distributed data. Differences between categorical variables were tested for significance using the Fisher’s exact test. A *P*-value <.05 was considered statistically significant.

## Results

### Study population

A total of 104 subjects were included (age 24.0 years [IQR 19.0–35.8 years], 43.3% men), of which 37 were preclinical *PKP2*-(L)PV carriers and 67 control subjects (Table 1), after 7 control subjects had been excluded because of the use of anti-arrhythmic medication (4 used class IV, 2 used class II, and 1 used class III antiarrhythmic medication). None of the *PKP2*-(L)PV carriers used antiarrhythmic medication at the time of ECG acquisition. All ECGs were evaluated through expert analysis and were unanimously described as normal according to current standards.^14^

**Table 1:** Baseline characteristics

Characteristic	All (n = 104)	Control (n = 67)	*PKP2*-(L)PV (n = 37)	*P*-value
Men (n)	45 (43.3%)	30 (44.8%)	15 (40.5%)	.686
Age (y)	24.0 [19.0–35.8]	23.0 [19.0–35.0]	24.0 [18.0–39.5]	.632
Heart rate (bpm)	68.2 ± 12.3	69.4 ± 12.1	66.2 ± 12.5	.209
QRS duration (ms)	91.3 ± 9.5	92.6 ± 9.4	88.9 ± 9.5	.054
QTc interval (ms)	408.0 ± 20.1	407.0 ± 19.4	409.8 ± 21.5	.487
Heart axis (°)	66.0 [44.0–78.8]	67.0 [31.0–77.0]	63.0 [47.0–79.8]	.806

Values are described as n (%), median (IQR) or mean ± SD. *P*-values are calculated between the control group and the carriers of a (likely) pathogenic variant in Plakophilin-2 (*PKP2*-(L)PV).

**Figure 2:**
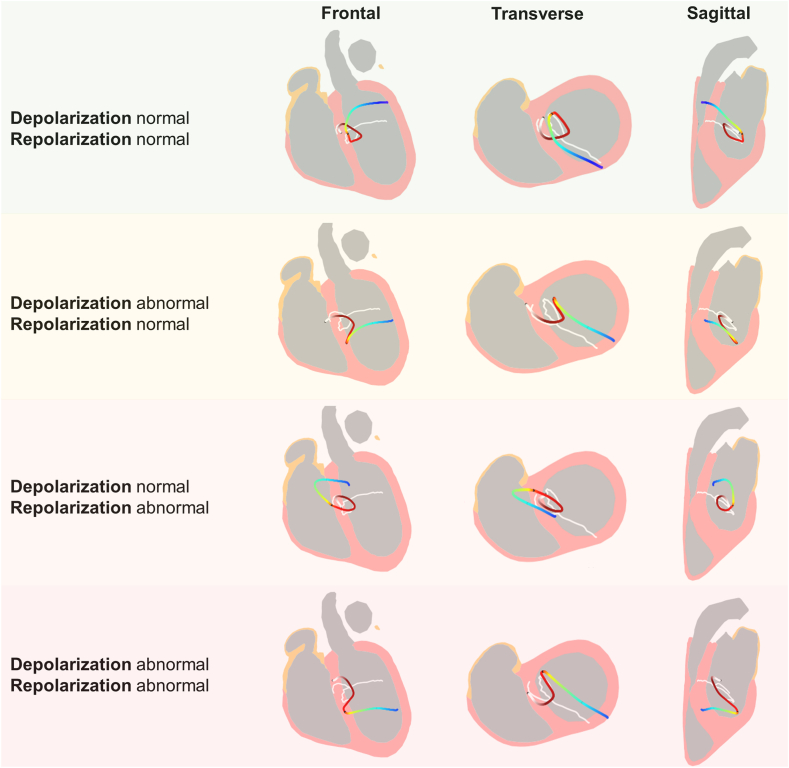
Depolarization and repolarization trajectories. Trajectories of 4 Plakophilin-2 (likely) pathogenic variant carriers for depolarization (*white* through *red*) and repolarization (*yellow* until *blue*). The median trajectory of the normal distribution is shown in *white*. Examples are shown of a trajectory that was normal for both depolarization and repolarization (*upper row*), abnormal depolarization and normal repolarization (*second row*), normal depolarization and abnormal repolarization (*third row*), and both abnormal depolarization and repolarization (*fourth row*).

The CineECG depolarization trajectory was classified as abnormal in 19 of 37 (51.4%) *PKP2*-(L)PV subjects (Table 2), the repolarization trajectory was abnormal in 9 of 37 (24.3%) *PKP2*-(L)PV carriers (Table 3). Seventeen (45.9%) carriers had both a normal depolarization and repolarization trajectory, 11 (29.7%) carriers had an abnormal depolarization trajectory but a normal repolarization trajectory, and 1 (2.7%) carrier had a normal depolarization trajectory with an abnormal repolarization trajectory. Eight (21.6%) carriers had both an abnormal depolarization trajectory and an abnormal repolarization trajectory. Examples of normal and abnormal de- and repolarization trajectories are shown in Figure 2.

**Table 2:** Depolarization trajectory characteristics

Characteristic	Controls (n = 67)	*PKP2*-(L)PV with normal depolarization trajectory (n = 18)	*PKP2*-(L)PV with abnormal depolarization trajectory (n = 19)	*P-*value controls vs *PKP2*-(L)PV with normal trajectory	*P-*value controls vs *PKP2*-(L)PV with abnormal trajectory	*P-*value normal vs abnormal trajectory from *PKP2*-(L)PV carriers
Direction left-right initial depolarization trajectory				.627	.064	.020
Far right (n)	3 (4.5%)	0 (0.0%)	3 (15.8%)			
Center (n)	59 (88.1%)	18 (100.0%)	13 (68.4%)			
Far left (n)	5 (7.5%)	0 (0.0%)	3 (15.8%)			
Direction inferior-superior initial depolarization trajectory				.463	.005	.010
Far inferior (n)	6 (9.0%)	0 (0.0%)	5 (26.3%)			
Center (n)	58 (86.6 %)	17 (94.4%)	10 (52.6%)			
Far superior (n)	3 (4.5%)	1 (5.6%)	4 (21.1%)			
Direction posterior-anterior initial depolarization trajectory				1.000	.020	.105
Far posterior (n)	0 (0.0%)	0 (0.0%)	0 (0.0%)			
Center (n)	2 (3.0%)	0 (0.0%)	4 (21.1%)			
Far anterior (n)	65 (97.0%)	18 (100.0%)	15 (78.9%)			
Direction left-right terminal depolarization trajectory				.661	.500	1.000
Far right (n)	22 (32.8%)	4 (22.2%)	4 (21.1%)			
Center (n)	39 (58.2%)	12 (66.7%)	12 (63.2%)			
Far left (n)	6 (9.0%)	2 (11.1%)	3 (15.8%)			
Direction inferior-superior terminal depolarization trajectory				.821	.074	.365
Far inferior (n)	12 (17.9%)	2 (11.1%)	3 (15.8%)			
Center (n)	20 (29.9%)	7 (38.9%)	11 (57.9%)			
Far superior (n)	35 (52.2%)	9 (50.0%)	5 (26.3%)			
Direction posterior-anterior terminal depolarization trajectory				.175	.786	.663
Far posterior (n)	29 (43.3%)	11 (61.1%)	10 (52.6%)			
Center (n)	27 (40.3%)	3 (16.7%)	6 (31.6%)			
Far anterior (n)	11 (16.4%)	4 (22.2%)	3 (15.8%)			

Values are described as n (%).

*PKP2*-(L)PV = (likely) pathogenic variant in Plakophilin-2.

**Table 3:** Repolarization trajectory characteristics

Characteristic	Controls (n = 67)	*PKP2*-(L)PV with normal repolarization trajectory (n = 28)	*PKP2*-(L)PV with abnormal repolarization trajectory (n = 9)	*P-*value controls vs *PKP2*-(L)PV with normal trajectory	*P-*value controls vs *PKP2*-(L)PV with abnormal trajectory	*P-*value normal vs abnormal trajectory from *PKP2*-(L)PV carriers
Direction left-right ST-segment				.612	.102	.302
Far right (n)	0 (0.0%)	0 (0.0%)	0 (0.0%)			
Center (n)	19 (28.4%)	6 (21.4%)	0 (0.0%)			
Far left (n)	48 (71.6%)	22 (78.6%)	9 (100.0%)			
Direction inferior-superior ST-segment				.653	1.000	.714
Far inferior (n)	36 (53.7%)	13 (46.4%)	5 (55.6%)			
Center (n)	31 (46.3%)	15 (53.6%)	4 (44.4%)			
Far superior (n)	0 (0.0%)	0 (0.0%)	0 (0.0%)			
Direction posterior-anterior ST-segment				.328	.701	1.000
Far posterior (n)	0 (0.0%)	0 (0.0%)	0 (0.0%)			
Center (n)	18 (26.9%)	11 (39.3%)	3 (33.3%)			
Far anterior (n)	49 (73.1%)	17 (60.7%)	6 (66.7%)			
Direction left-right T-wave				1.000	.477	1.000
Far right (n)	0 (0.0%)	0 (0.0%)	0 (0.0%)			
Center (n)	4 (6.0%)	2 (7.1%)	1 (11.1%)			
Far left (n)	63 (94.0%)	26 (92.9%)	8 (88.9%)			
Direction inferior-superior T-wave				.771	.668	.620
Far inferior (n)	55 (82.1%)	24 (85.7%)	7 (77.8%)			
Center (n)	12 (17.9%)	4 (14.3%)	2 (22.2%)			
Far superior (n)	0 (0.0%)	(0.0%)	0 (0.0%)			
Direction posterior-anterior T-wave				1.000	1.000	1.000
Far posterior (n)	0 (0.0%)	0 (0.0%)	0 (0.0%)			
Center (n)	39 (58.2%)	17 (60.7%)	5 (55.6%)			
Far anterior (n)	28 (41.8%)	11 (39.3%)	4 (44.4%)			

Values are described as n (%).

*PKP2*-(L)PV = (likely) pathogenic variant in Plakophilin-2.

### Depolarization trajectory

The CineECG depolarization trajectory of the control subjects typically started left of the septum and then moved toward the right, representing septal activation. It then moved in the direction of the apex, and the latest activation was directed toward the left basal area of the heart. The depolarization phase usually resembled an O- or U-shape, as seen from the transverse plane.

Although the direction of the initial depolarization trajectory along the left-right axis stayed mainly in the center in controls and in *PKP2*-(L)PV carriers with a normal depolarization trajectory, the direction was significantly different in those with an abnormal depolarization trajectory (*P* = .020). The direction could be either more toward the far right or far left in these subjects. In the inferior-superior axis, the direction of those with an abnormal depolarization trajectory was also significantly different from *PKP2*-(L)PV carriers with a normal trajectory (*P* = .010) and controls (*P* = .005). Although in the posterior-anterior direction, the initial vector was directed toward the anterior in controls and *PKP2*-(L)PV carriers with a normal trajectory, the direction of those with an abnormal trajectory was significantly different to controls (*P* = .020), as a direction that stayed in the center was more frequently observed (Table 2). Although it just failed to reach significance, the direction of the terminal depolarization trajectory along the inferior-superior axis was more frequently observed to be in the center in those with an abnormal depolarization trajectory, whereas in controls it was most frequently directed toward the superior.

### Repolarization trajectory

A normal CineECG repolarization trajectory of the control subjects was usually directed from base to apex/left lateral wall in a relatively straight line. The CineECG repolarization trajectories were very similar in direction between the abnormal and normal *PKP2*-(L)PV carriers and between the control group, as none of the directions of the repolarization trajectory differed significantly between the groups (Table 3).

### Baseline clinical evaluation

Mild abnormalities were identified with Holter and/or imaging in 20 of 37 *PKP2*-(L)PV subjects (54.1%). Nineteen carriers (51.4%) had an abnormality as identified by imaging, whereas Holter monitoring revealed a minor abnormality in 6 carriers (16.2%) (Table 4). In the group with normal CineECG depolarization and repolarization trajectories, 8 of 17 carriers (47.1%) did not show any mild abnormality on Holter and/or imaging, whereas 11 of 20 subjects (55.0%) with an abnormal depolarization and/or repolarization trajectory had a mild abnormality on imaging and/or Holter.

**Table 4:** Clinical characteristics and follow-up

	Depolarization and repolarization normal (n = 17)	Depolarization abnormal and repolarization normal (n = 11)	Depolarization normal and repolarization abnormal (n = 1)	Depolarization and repolarization abnormal (n = 8)
**Mild abnormality at baseline**				
Imaging and Holter (n)	2 (11.8%)	2 (18.2%)	0 (0.0%)	1 (12.5%)
Imaging (n)	7 (41.2%)	4 (36.3%)	0 (0.0%)	3 (37.5%)
Holter (n)	0 (0.0%)	1 (9.1%)	0 (0.0%)	0 (0.0%)
No mild abnormality (n)	8 (47.1%)	4 (36.4%)	1 (100.0%)	4 (50.0%)
**Outcomes during follow-up**				
Palpitations (n)	2 (11.8%)	1 (9.1%)	0 (0.0%)	1 (12.5%)
Syncope (n)	0 (0.0%)	0 (0.0%)	0 (0.0%)	0 (0.0%)
Ventricular tachycardia (n)	1 (5.9%)	1 (9.1%)	0 (0.0%)	0 (0.0%)
ICD implantation (n)	1 (5.9%)	0 (0.0%)	0 (0.0%)	0 (0.0%)
TFC acquirement (n)	5 (29.4%)	4 (36.4%)	0 (0.0%)	1 (12.5%)
No follow-up available (n)	6 (35.3%)	1 (9.1%)	0 (0.0%)	1 (12.5%)

The groups are based on the classification of the CineECG trajectories. Values are described as n (%).

ICD = implantable cardioverter defibrillator; TFC = Task Force Criteria.

### Follow-up clinical evaluation

Follow-up was available in 29 subjects. The median follow-up duration was 4.1 years (IQR 2.1–8.0 years). During follow-up, 10 (34.5%) subjects acquired a new TFC (Table 4) during a median follow-up duration of 6.9 years (IQR 4.6–11.2 years). Out of those 10 subjects, 5 carriers had an abnormal CineECG depolarization and/or repolarization trajectory, and 5 subjects had a normal de- and repolarization trajectory. The median time until TFC fulfillment was 1.3 years (IQR 0.7–4.9 years) in the group with an abnormal de- and/or repolarization trajectory. The time until TFC fulfillment for the 5 subjects with a normal de- and repolarization trajectory was 4.4 years (IQR 3.0–6.5 years). One subject with a normal de- and repolarization trajectory received an implantable cardioverter-defibrillator 8.8 years after baseline for primary prevention. Ultimately, the same subject developed VT, which first occurred 18.3 years after baseline evaluation. One other subject also developed VT. This subject had an abnormal depolarization trajectory and a normal repolarization trajectory. This VT occurred 9.4 years after baseline.

The other 19 subjects (65.5%) did not receive any TFC during follow-up. Thirteen (68.4%) of these subjects had an abnormal depolarization and/or repolarization trajectory. The median follow-up duration in the group without new TFC was 3.8 years (IQR 2.0–5.8 years). Specifically, the median follow-up in the 6 subjects with normal CineECG trajectories was 3.6 years (IQR 1.8–7.4 years), and 3.8 years (IQR 2.0–5.2 years) in the 13 carriers with an abnormal CineECG trajectory.

Symptoms of palpitations were present in 4 subjects. In the group with a normal de- and repolarization trajectory, 2 subjects developed symptoms of palpitations after 7.2 and 9.0 years, respectively. One subject with palpitation complaints who had an abnormal depolarization and repolarization trajectory, already had these complaints at baseline, and the other subject developed palpitations after 6.8 years. None of the subjects had syncope during follow-up.

## Discussion

The most important finding of this study is that the novel CineECG technique non-invasively identified abnormal depolarization and repolarization in over half of preclinical *PKP2*-(L)PV carriers, with an ECG that was judged as normal according to the current standards. Abnormalities in the depolarization trajectory were more common than abnormalities in the repolarization trajectory. These results indicate that subtle electrical abnormalities already exist in a considerable number of preclinical *PKP2*-PV carriers, which would otherwise not be detected on the 12-lead ECG.

### Depolarization abnormalities

The CineECG trajectory showed that the initial ventricular electrical activation was different in the group with an abnormal depolarization trajectory. It is possible that the described pathophysiological mechanisms in *PKP2-*(L)PV carriers, such as disrupted cell-to-cell adhesion and the infiltration of fibrofatty tissue,^15^ have already begun across the right ventricle even in these preclinical carriers; however, have (largely) remained undetected by our current standard diagnostic modalities. Early, subclinical fibrosis might result in a zig-zag course of activation, leading to conduction delay in the right ventricle which results in the abnormal direction of the depolarization trajectory.^16^ Although invasive studies have been performed in definite ACM, which have detected low amplitudes in the RV outflow tract, subtricuspid area, RV free wall, and the RV apex,^17^ very little is known about possibly abnormal electrical cardiac activity in preclinical *PKP2*-(L)PV carriers. The results of this study indicate that these preclinical subjects can have electrical abnormalities, although the clinical implications are still unclear.

### Repolarization abnormalities

The CineECG repolarization trajectory was abnormal in a minority of the *PKP2*-(L)PV carriers. Still, the directions were similar between the normal and abnormal repolarization trajectories. Repolarization abnormalities are characteristic of ACM, with T-wave inversions being the most frequently observed ECG abnormality.^11^ Although none of the subjects had any T-wave inversions, the repolarization sequence was still classified as abnormal in 8 subjects. As the direction of the repolarization trajectory was similar between controls and all *PKP2*-(L)PV carriers, those with an abnormal repolarization trajectory likely had a repolarization trajectory that was mostly parallel to the normal distribution, although just outside this distribution for more than 5% of the trajectory.

### Clinical evaluation

Half of the subjects (50%) who ultimately progressed toward new TFC had an abnormal CineECG trajectory. The median time until TFC fulfillment in those with abnormal de- and/or repolarization was much shorter (approximately 1 year) than those with a normal de- and repolarization who ultimately progressed toward TFC (over 4 years). In the group who ultimately did not receive a new TFC, 68.4% also had an abnormal de- and/or repolarization trajectory. However, the median follow-up duration in these subjects (3.8 years) was slightly shorter than the median time until TFC fulfillment (4.4 years). It is possible that there was not yet enough follow-up time for TFC development, although it is also possible that these subjects will not progress toward TFC, and that the abnormal patterns are not necessarily related to disease progression, but are simply a characteristic within these *PKP2*-(L)PV carriers.

There was no complete overlap in the abnormalities detected by CineECG and by the conventional modalities. It has been described that electrical abnormalities precede structural abnormalities, whereas the opposite—structural abnormalities preceding electrical abnormalities—has also been reported.^8^^,^^13^ There might therefore always be some discrepancy between the electrical and structural abnormalities found in *PKP2-*(L)PV carriers.

### Non-invasive ECG modalities in early detection of ACM

This study confirms the findings of previous attempts to detect early signs of electrical abnormalities in preclinical *PKP2* and other desmosomal variant carriers. Body surface potential mapping and ECG imaging have revealed abnormal patterns in de- and repolarization.^18^, ^19^, ^20^, ^21^ Kommata et al^19^ also found abnormal activation patterns using ECG imaging both in the early and late depolarization in preclinical desmosomal variant carriers. They describe abnormal locations of the earliest activation of the RV in the basal segments of the RV anterior and inferior wall, and in the RV free wall. Kloosterman et al^18^ specifically looked into *PKP2-*PV carriers. They describe 4 different types of abnormal activation patterns in preclinical variant carriers. The findings of these 2 studies are in concordance with the results of this study, where abnormal directions were not found in 1 single axis and in 1 single direction, but rather across multiple axes and multiple directions. This implies that although abnormal depolarization occurs in preclinical variant carriers, no uniform pattern can be observed that applies to all carriers.

Kloosterman et al^18^ also described abnormal repolarization patterns in preclinical *PKP2*-PV carriers. They observed a slightly changed location of the maximum amplitudes during repolarization on the body surface map, being located more central-inferior in preclinical *PKP2*-PV carriers, as opposed to left-inferior in control subjects. In 1 case, a second maximum was observed at infero-posterior placed leads. In our study, no distinct differences were observed in the direction of the repolarization trajectories between controls and *PKP2*-(L)PV carriers; the general direction of the repolarization trajectory was directed left-inferior. A slight change in direction, as described in the study of Kloosterman et al,^18^ is likely overlooked, as the directions were categorized into 3 directions, which only covered the center, far left-right, far inferior-superior, and far posterior-anterior directions.

The amount of abnormal depolarization and repolarization patterns observed in this study is similar to the previous studies on preclinical variant carriers. Abnormal depolarization patterns were previously reported in 57%–60% of preclinical carriers,^18^^,^^19^ whereas we report abnormal depolarization in 51% of *PKP2*-(L)PV carriers. Abnormal repolarization was reported in 16%–25% in previous studies,^18^^,^^21^ which is similar to the 24% that was found in this study. The consistency of these observations across multiple studies supports the validity of these findings, and points toward a subgroup of preclinical mutation carriers with abnormal electrical activation of the right ventricle, whereas they are otherwise considered to be in a preclinical stage of disease. Presumably, these subjects carry a higher risk of developing disease. Although these previous studies relied on extensive electrode configurations, this study was able to demonstrate similar results while only using the 12-lead configuration.

## Limitations

A limitation of this study is the small sample size. A larger cohort might have been created by including other desmosomal variants, however, a gene-specific approach will be better clinically applicable to *PKP2*-(L)PV carriers.^22^ Limitations of this study also include the relatively short follow-up period. Longer follow-up in a larger cohort is warranted to truly understand the clinical implications of these results, and if these subjects are at a higher risk to develop phenotypical signs of disease.

A more ideal control group for this study might have consisted of asymptomatic, genotype-negative family members who had undergone cardiac screening and were confirmed to have no structural or electrical abnormalities. In our database, however, this particular group is very small since genotype-negative family members have been reassured, and usually have not undergone extensive screening including imaging. Therefore, we used patients with atrioventricular nodal reentrant tachycardia and normal imaging as controls. As atrioventricular nodal reentrant tachycardia is a supraventricular arrhythmia that is not expected to affect ventricular depolarization or repolarization, this group can be considered a reasonable alternative for defining normal CineECG trajectories.

## Conclusion

This study demonstrated that analysis of cardiac 3D electrical activity trajectories with CineECG detected abnormal electrical activity in more than half of preclinical *PKP2*-(L)PV carriers with an apparently normal ECG. This novel diagnostic tool could provide a way to unveil subtle and early electrical abnormalities already hidden in the 12-lead ECG that would otherwise not be easily detected. Future research should focus on the added value of CineECG in the risk prediction of disease expression in PV carriers.

## References

[1] Boonstra M.J., Brooks D.H., Loh P., van Dam P.M. (2022). CineECG: a novel method to image the average activation sequence in the heart from the 12-lead ECG. Comput Biol Med.

[2] Boonstra M., Kloosterman M., van der Schaaf I., Roudijk R., van Dam P., Loh P. (2023). ECG-based techniques to enhance clinical practice in cardiac genetic disease management. J Electrocardiol.

[3] Boonstra M.J., Hilderink B.N., Locati E.T., Asselbergs F.W., Loh P., van Dam P.M. (2021). Novel *CineECG* enables anatomical 3D localization and classification of bundle branch blocks. EP Europace.

[4] van der Schaaf I., Kloosterman M., Boonstra M.J., van Dam P.M., Gorgels A.P.M. (2023). CineECG illustrating the ventricular activation sequence in progressive AV-junctional conduction block. J Electrocardiol.

[5] van der Schaaf I., Kloosterman M., Gorgels A.P.M., Loh P., van Dam P.M. (2024). CineECG for visualization of changes in ventricular electrical activity during ischemia. J Electrocardiol.

[6] van Dam P.M., Locati E.T., Ciconte G. (2020). Novel CineECG derived from standard 12-lead ECG enables right ventricle outflow tract localization of electrical substrate in patients with Brugada syndrome. Circ Arrhythm Electrophysiol.

[7] Frosted R., Paludan-Müller C., Vad O.B. (2023). CineECG analysis provides new insights into Familial ST-segment Depression Syndrome. Europace.

[8] Krahn A.D., Wilde A.A.M., Calkins H. (2022). Arrhythmogenic right ventricular cardiomyopathy. JACC Clin Electrophysiol.

[9] Marcus F.I., McKenna W.J., Sherrill D. (2010). Diagnosis of arrhythmogenic right ventricular cardiomyopathy/dysplasia. Eur Heart J.

[10] Towbin J.A., McKenna W.J., Abrams D.J. (2019). HRS expert consensus statement on evaluation, risk stratification, and management of arrhythmogenic cardiomyopathy. Heart Rhythm.

[11] Corrado D., Basso C., Judge D.P. (2017). Arrhythmogenic cardiomyopathy. Circ Res.

[12] Richards S., Aziz N., Bale S. (2015). Standards and guidelines for the interpretation of sequence variants: a joint consensus recommendation of the American College of Medical Genetics and Genomics and the Association for Molecular Pathology. Genet Med.

[13] Mast T.P., Taha K., Cramer M.J. (2019). The prognostic value of right ventricular deformation imaging in early arrhythmogenic right ventricular cardiomyopathy. JACC Cardiovasc Imaging.

[14] Macfarlane P.W., Veitch L.T., Van Oosterom A., Pahlm O., Kligfield P., Janse M., Camm J. (2010). Comprehensive Electrocardiology.

[15] Costa S., Cerrone M., Saguner A.M., Brunckhorst C., Delmar M., Duru F. (2021). Arrhythmogenic cardiomyopathy: an in-depth look at molecular mechanisms and clinical correlates. Trends Cardiovasc Med.

[16] De Bakker J.M.T., Van Rijen H.M.V. (2006). Continuous and discontinuous propagation in heart muscle. J Cardiovasc Electrophysiol.

[17] Saguner A.M., Lunk D., Mohsen M. (2023). Electroanatomical voltage mapping with contact force sensing for diagnosis of arrhythmogenic right ventricular cardiomyopathy. Int J Cardiol.

[18] Kloosterman M., Boonstra M.J., Roudijk R.W. (2023). Body surface potential mapping detects early disease onset in plakophilin-2 -pathogenic variant carriers. Europace.

[19] Kommata V., Sciaraffia E., Blomström-Lundqvist C. (2023). Epicardial conduction abnormalities in patients with Arrhythmogenic Right Ventricular Cardiomyopathy (ARVC) and mutation positive healthy family members–a study using electrocardiographic imaging. PLoS One.

[20] Kommata V., Elshafie M., Sciaraffia E., Perez M., Augustine R., Blomström-Lundqvist C. (2021). QRS dispersion detected in ARVC patients and healthy gene carriers using 252-leads body surface mapping: an explorative study of a potential diagnostic tool for arrhythmogenic right ventricular cardiomyopathy. Pacing Clin Electrophysiol.

[21] Kommata V., Sciaraffia E., Blomström-Lundqvist C. (2022). Repolarization abnormalities unmasked with a 252-lead BSM system in patients with ARVC and healthy gene carriers. Pacing Clin Electrophysiol.

[22] Murray B., James C.A. (2022). Genotype–phenotype correlates in arrhythmogenic cardiomyopathies. Curr Cardiol Rep.

