# Supplementary material for: CineECG detects abnormal electrical activity in the 12-lead ECG of preclinical plakophilin-2 variant carriers
Source: Heart Rhythm O2. 2025 Aug 8;6(11):1786–95. doi: 10.1016/j.hroo.2025.07.105 (PMC12675129; doi:10.1016/j.hroo.2025.07.105)
Supplement: Supplementary Material [file mmc1.docx]

***Supplemental Figure 1:*** *A hypothetical direction is shown from an anterior view (left image) and a left sagittal view (right image). The directions from each part of the CineECG trajectory (initial depolarization, terminal depolarization, ST-segment and T-wave) are categorized to be in the center (between -0.5 and 0.5) or the far end of each axis (>0.5 or <-0.5). The starting point of this part of the trajectory is set to x-y-z coordinates 0,0,0. In this particular example, the direction would be categorized to be in the center of the left-right axis (end of the vector is at -0.3 for this axis), far superior (end of vector is at 0.6 for this axis) and far posterior (end of vector is at -0.6 for this axis).*
